# Device Thrombogenicity Emulation: A Novel Method for Optimizing Mechanical Circulatory Support Device Thromboresistance

**DOI:** 10.1371/journal.pone.0032463

**Published:** 2012-03-02

**Authors:** Gaurav Girdhar, Michalis Xenos, Yared Alemu, Wei-Che Chiu, Bryan E. Lynch, Jolyon Jesty, Shmuel Einav, Marvin J. Slepian, Danny Bluestein

**Affiliations:** 1 Department of Biomedical Engineering, Stony Brook University, Stony Brook, New York, United States of America; 2 Department of Hematology, Stony Brook University, Stony Brook, New York, United States of America; 3 MicroMed Cardiovascular Inc., Houston, Texas, United States of America; 4 Sarver Heart Center, University of Arizona, Tucson, Arizona, United States of America; Medizinische Hochschule Hannover, Germany

## Abstract

Mechanical circulatory support (MCS) devices provide both short and long term hemodynamic support for advanced heart failure patients. Unfortunately these devices remain plagued by thromboembolic complications associated with chronic platelet activation – mandating complex, lifelong anticoagulation therapy. To address the unmet need for enhancing the thromboresistance of these devices to extend their long term use, we developed a universal predictive methodology entitled Device Thrombogenicity Emulation (DTE) that facilitates optimizing the thrombogenic performance of any MCS device – ideally to a level that may obviate the need for mandatory anticoagulation.

DTE combines *in silico* numerical simulations with *in vitro* measurements by correlating device hemodynamics with platelet activity coagulation markers – before and after iterative design modifications aimed at achieving optimized thrombogenic performance. DTE proof-of-concept is demonstrated by comparing two rotary Left Ventricular Assist Devices (LVADs) (DeBakey vs HeartAssist 5, Micromed Houston, TX), the latter a version of the former following optimization of geometrical features implicated in device thrombogenicity. Cumulative stresses that may drive platelets beyond their activation threshold were calculated along multiple flow trajectories and collapsed into probability density functions (PDFs) representing the device ‘thrombogenic footprint’, indicating significantly reduced thrombogenicity for the optimized design. Platelet activity measurements performed in the actual pump prototypes operating under clinical conditions in circulation flow loops – before and after the optimization with the DTE methodology, show *an order of magnitude* lower platelet activity rate for the optimized device. The robust capability of this predictive technology – demonstrated here for attaining safe and cost-effective pre-clinical MCS thrombo-optimization – indicates its potential for reducing device thrombogenicity to a level that may significantly limit the extent of concomitant antithrombotic pharmacotherapy needed for safe clinical device use.

## Introduction

Congestive heart failure, the final common pathway of all forms of heart disease, has reached epidemic proportions in the United States and in many nations around the world. Over 5.7 million patients suffer from heart failure in the US, with the number of patients expected to grow by 50% over the next 15 years [1]. In the spectrum of heart failure severity, approximately 100,000 patients a year are afflicted with AHA/ACC Class D, NYHA Class IV disease. These patients have such reduced ventricular systolic function that they need a pump, either as an assist device or as a replacement. Mechanical circulatory support (MCS) devices, i.e. ventricular assist devices (VADs) and the total artificial heart (TAH), offer this pump therapy, either as a bridge to eventual heart transplantation or as an alternative to transplantation [2]. While life saving, the current devices have many limitations, including the propensity for thrombosis and thromboembolism, often introducing clinical sequelae that further increases the healthcare burden to the patient and society. Recipients of VADs and mechanical heart valves (MHVs) are often faced with the requirement of a lifetime of complex and risky anticoagulation regimens. Although the current generation of MCS devices shows a vast improvement in hemodynamic performance over their predecessors, reducing flow-induced thrombogenicity still remains a major challenge and this could drastically change through development of a new generation of devices that are optimized to be thromboresistant (i.e., with a reduced need for antithrombotic therapy).

The high incidence of thromboembolic events in MCS devices occurs largely due to non-physiological flow past constricted geometries within a device, where platelets, the principal cellular clotting elements in blood, are exposed to elevated shear stresses and exposure times [3]. Platelets activated by this mechanism are considered one of the major culprits in attempting to modulate the hemostatic response in MCS devices, even in the absence of contact activation and/or chemical agonists [4]. Although the threshold for shear induced platelet activation has been established based on constant stress experiments (i.e., Hellum's criterion [4], [5]), it should be noted that under physiological conditions of flow through devices, platelets are exposed to varying shear stress levels, turbulent stresses [6], longer residence times in pathological flow regions (hot-spots), as well as repeated passages through the device that may precipitate activation [3], aggregation, and free emboli formation.

Presently hemodynamic studies employed for device optimization during the R&D stage have significant limitations as follows: (a) They utilize experimental flow visualization and/or traditional computational fluid dynamics (CFD) mostly concentrating on mapping regions of high shear stresses with no direct correlation to thrombogenic markers. (b) Most of them do not resolve the intricate, small-scale, flow phenomena and their interaction with blood-borne particulates such as platelets which initiate device thrombogenicity (i.e., drive platelets past the activation threshold as discussed above). (c) These methods do not map specific stress loading histories that platelets experience along their corresponding flow trajectories. As such, they cannot be utilized for identifying and eliminating thrombogenic hot-spots within the devices and are therefore mostly applied *ad hoc*. (d) There is no iterative synergy between numerical and experimental methods and this significantly limits device thromboresistance optimization.

To address the above limitations, we have developed a methodology – Device Thrombogenicity Emulation (DTE) that bridges this gap by integrating the pertinent hemodynamics with the corresponding thrombogenic markers – facilitating for the first time an *actual optimization* of the thromboresistance performance. DTE offers a paradigm shift by integrating state-of-the-art numerical and experimental approaches *in silico* and *in vitro* (rather than *in vivo*). This facilitates optimizing the device during the R&D phase. The optimization process is performed first *in silico* – in the modeling domain (in which virtual design modifications are examined) – followed by experimental emulation of the device specific stress loading histories (waveforms) *in vitro* in which the resultant platelet activity is measured. The process is iterated till optimization (i.e., thrombogenicity minimization) is achieved. The optimized design prototype is built and tested by comparing its thrombogenic performance to that before the optimization. A detailed description of the methodology appears in Materials and Methods.

To demonstrate a proof-of-concept of the DTE methodology applied to an actual device, we took and redesigned a representative VAD (a rotary blood pump design) that had checkered history of thrombogenic complications which affected the overall clinical results [7], [8]. With DTE, we show that an order of magnitude reduction in thrombogenicity can be achieved with this VAD, and that the redesigned prototype may be suitable for implantation with the possibility that its recipients may benefit from a significantly reduced need for pharmacologically complex antithrombotic therapy.

## Materials and Methods

### Device Thrombogenicity Emulation (DTE)

**Figure 1:**
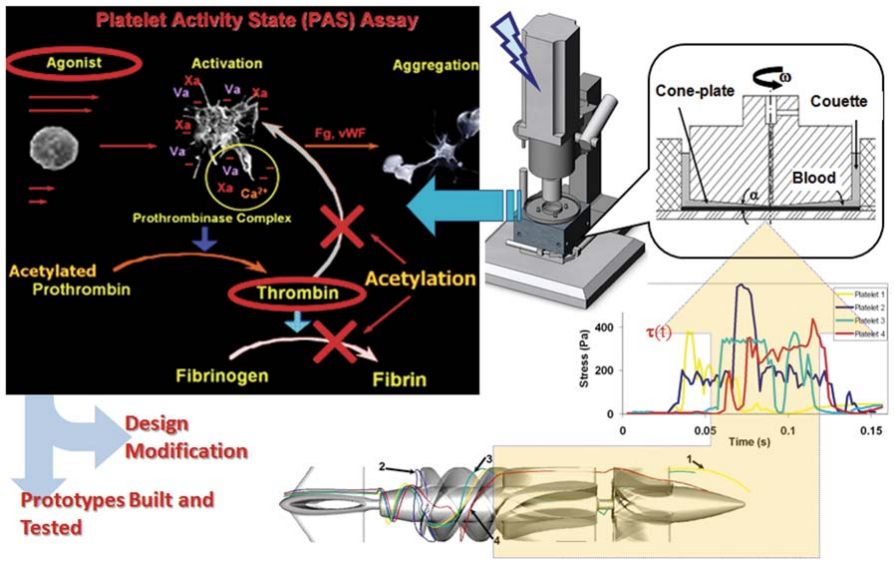
Schematic of the Device Thrombogenicity Emulation (DTE). (bottom-left) Representative platelet trajectories in the flow field of a ventricular assist device (debakey VAD); (bottom-right) Emulation of stress-loading history of typical platelet trajectories in the hemodynamic shearing device (HSD); (top-right) Computer-controlled HSD where platelets are exposed to uniform shear stress; (top-left) Principle of the modified prothrombinase assay used to measure the activity state of platelets sampled from the HSD.

A schematic of DTE is shown in Figure 1, illustrating application to a ventricular assist device (VAD). The optimization process is performed first *in silico* – in the modeling domain – followed by experimental emulation of the device specific stress loading histories (waveforms) in a Hemodynamic Shearing Device (HSD; Figure 1 – top-right) [9]) where device specific effects on platelet activation are measured with a modified prothrombinase assay (Figure 1, top-left) [10]. The features and advantages of this assay are described in the Experimental Flow Loop and Platelet Activation Measurement section. Flow past the VAD is modeled with a high fidelity two-phase FSI (fluid-structure interaction) simulation resolving all components of the stress tensor that are relevant to flow-induced thrombogenicity and subsequently tracking down and capturing the loading history of platelets in the flow field along trajectories that may drive them beyond the activation threshold. An example of platelet trajectories in the flow field of a VAD is shown in Figure 1 (bottom-left) and stress-loading histories of these four platelet trajectories (computed with the combined effect of shear stress and exposure time [3], [5], [11]) is shown in Figure 1 (bottom-right). Typically, several thousand of such loading histories are collapsed into a quantitative probability density function (PDF) – a thrombogenic footprint of the device [12], [13] that maps where the cumulative stress of each trajectory will end up in the distribution of the stress accumulation range of the device. To validate this *in silico* approach experimentally, extreme cases of platelet stress loading trajectories termed as hot-spot trajectories are extracted and tested in the HSD and platelet activity is quantified for these trajectories with a modified prothrombinase assay [10] (Figure 1; top-left). Virtual design modifications to the original device geometry or its components can then be performed and above *in silico* and experimental process iterated in the virtual domain to map the least thrombogenic footprint of the redesigned device. The prototype device design is then frozen and prototypes are manufactured according to their optimized specifications. Comparative thrombogenicity experiments of the original and optimized prototypes (whole devices) are then performed *in vitro* to confirm that the reduction in thrombogenicity with DTE methodology was achieved in the optimized device prototype.

### Blood Pumps

**Figure 2:**
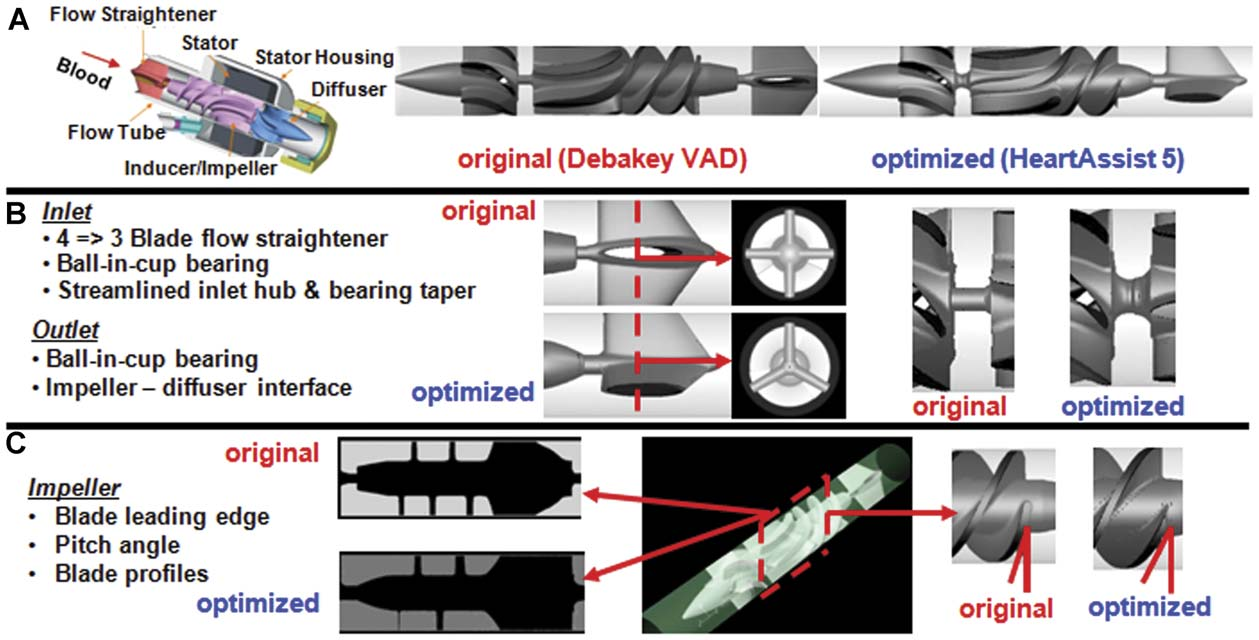
Design changes in Debakey VAD for reducing thrombogenicity. (**A**) (left) Components of the Original Debakey VAD; (right) Original (Debakey) and Optimized (HeartAssist 5) VAD prototypes. (**B**) Design differences in the inlet and outlet region are illustrated for both VADs. (**C**) Design differences in the impeller region are illustrated for both VADs.

We tested two rotary pump device prototypes – the DeBakey™ VAD and the redesigned optimized version of this device – the HeartAssist 5™. Both prototypes were provided by the same manufacturer (Figure 2A; top-right; MicroMed Cardiovascular Inc., Houston, TX) for experimental measurements in addition to the 3D prototype geometries for numerical simulations. The basic pump features and control unit are described in detail elsewhere [14]. Briefly, the main components of the DeBakey VAD (Figure 2A; top-left) – include a stationary flow straightener, an inducer-impeller with an integrated electric motor rotor (moving unit), a stationary diffuser, and motor stator housing. The following design features, illustrated in Figure 2B and Figure 2C, were modified between the original and the optimized VAD: (A) Inlet flow straightener vanes: Four orthogonal vanes in the original design were replaced with three vanes, 120° apart. (B) Inlet and outlet hub and bearing taper: The back-step front hub between the flow straightener and the inducer-impeller was tapered and streamlined as indicated to eliminate flow stagnation and backflow, and the outlet hub was retrofitted with ball-in-cup bearing to minimize recirculation in the impeller-diffuser interface. (C) Impeller: The impeller blade leading edge, profiles, length, pitch, and pitch-angle (which direct the flow into the inducer-impeller) were modified.

### Computational Grid and Simulation Procedure

**Figure 3:**
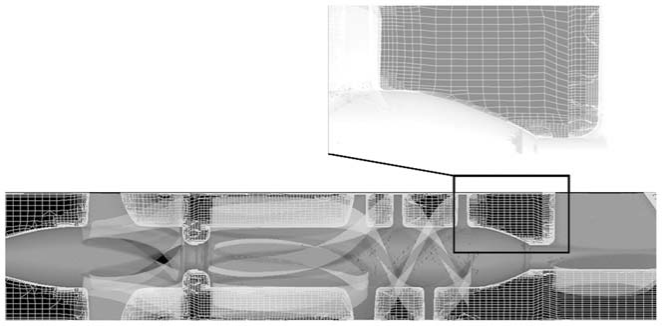
Hexahedral mesh configurations (∼3.7 million elements) for FSI simulations generated with HEXPRESS. Note refinement at the boundary layers (*inset*).

Fluid structure Interaction (FSI) simulations were conducted using Unsteady Reynolds Averaged Navier-Stokes (URANS) for 25 impeller revolutions to solve blood flow through both VAD geometries. Blood was modeled as a two-phase fluid [15] (platelets considered as neutrally buoyant solid spherical particles of 3 µm diameter) with a density of 1,081 kg/m^3^ and a viscosity of 0.0035 kg/m.s. A rotational speed of 9,500 rpm and a flow rate of 4 L/min, corresponding to most commonly encountered clinical operating conditions, were used for both simulations. Highly resolved hexahedral computational grids (3.68 million elements) were generated in HEXPRESS (Numeca International, CA) to achieve a boundary layer sublayer (y+ = 1; corresponding to the nearest boundary element height of 10 µm) necessary for the two equation k-ω [16] turbulence model as part of the URANS. Details of the mesh are shown in Figure 3 with the finely resolved boundary layer shown in *inset*. The Fluent CFD solver (Ansys Inc., Lebanon, NH) was used for this calculation and an optimized time step of 7.0×10^−5^ s was selected per iteration and corresponds to a 4-degree change in the rotor position. 4,000–5,000 platelets were injected from a plane at the tip of the inlet flow straightener. The simulation approach is further described in detail in our previous publications [12], [13].

### Stress Accumulation (SA) Model

Stress loading history of the seeded platelets along trajectories was computed by incorporating the combined effect of stress and exposure time [5]. The stress tensor was extracted from the simulations along the corresponding platelet trajectories and rendered into a scalar stress value, as previously described [12], [13],
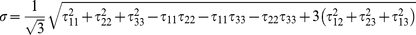
(1)so that both viscous and turbulent stresses (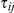) are considered in the SA formulation. A linear product of instantaneous values of the stress, 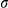, and exposure time, 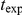 was assumed [3], [13] for the stress accumulation (SA) and a summation of this instantaneous product values is carried out according to:
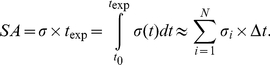
(2)where 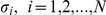, is the nodal scalar value extracted from the total stress tensor described above, Δ*t* is the corresponding time step between successive nodal points, and the resulting SA units are in Pa.s. While other models of stress accumulation argue for various power law combinations in the form of 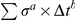, this model seems to predict platelets stress accumulation in devices better, and was recently validated with platelet activation measurements in channels [17], as well as by our previous work [3], [13].

### Probability Density Function (PDF) of Stress Accumulation

To account for the large number of platelets flowing along multiple trajectories in a complex flow field such as flow within a VAD, we have used a robust statistical technique (applied by us recently to compare and optimize MHV designs [12], [13]) to collapse this information into a quantity that could be used for comparing the thrombogenic potential of different VAD designs. This approach computes the statistical distribution of stress accumulation (based on SA ranges) along the multiple trajectories (the probability density function – PDF) and is therefore used as a surrogate for evaluating the overall thrombogenic potential of each VAD (a thrombogenic ‘footprint’ of the VAD design). To compare the statistical distribution of stress accumulations of different platelet populations that were used in the various simulations (4,000–5,000), while guaranteeing that the percentage activation is independent of variations in the number of seeded particles and spatiotemporal variations, we have interpolated between the smaller and larger populations' statistical distributions by applying bootstrapping statistics [12], [13] to guarantee that PDFs from the different population sizes are compatible and comparable. The mean value and standard deviation for each statistical distribution (PDF) for both the overall VAD designs and in regions of interest specific to design changes: (A) Inlet flow straightener (vanes), (B) Inlet hub and bearing taper, (C) Impeller, and (D) Impeller-shroud near wall region and tip-shroud gap clearance, are reported in the results.

### Experimental Flow Loop & Platelet Activation Measurement

**Figure 4:**
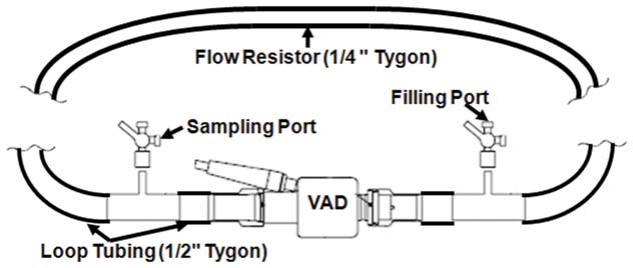
Flow loop schematic for conducting platelet activation measurements in the two VADs. A flow-resistor of identical lengths is used for both VADs to produce physiological pressure rise across the VADs.

A flow-loop that generates physiological range of cardiac outputs (3–6 L/min) as a function of pump speed (RPM) and pressure (ΔP) was built (Figure 4). The flow loop consists of two Tygon R3603 tubes – a 0.5 inch ID flow tube connected to the VAD and a 0.25 inch ID flow resistor. A cardiac output of 4 L/min which represents the average physiological condition was chosen. This corresponded to an impeller speed of 9500 RPM for both pumps, which is within the clinical operating range for continuous flow VADs.

Citrated blood (120 ml) was obtained from healthy adult volunteers (n = 10) who have abstained from all medication, including aspirin, for at least two weeks prior to blood donation, after obtaining written and informed consent (protocol approved by Stony Brook University IRB) prior to venipuncture. Gel-filtered platelets were prepared and diluted (15×10^6^/ml) in modified Tyrode's buffer. Red blood cells were purified with Histopaque 1119 (Sigma, St. Louis, MO) and diluted to 800×10^6^/ml in modified Tyrode's buffer. Platelets or platelets with red blood cells (120 ml) were recirculated in a physiological flow loop for 30 min that generates a pressure rise across the pump of ∼70–80 mm Hg as described above. Platelet activation was measured with a modified prothrombinase Platelet Activity State (PAS) assay [9], [10], from timed samples at t = 0, 10, 20, 30 min. The key advantage of using acetylated prothrombinase in our assay is that an acetylated form of thrombin is generated – which cleaves the chromogenic substrate identically to native thrombin – but is otherwise incapable of causing any feedback activation into the coagulation cascade (Figure 1 – top-left). Therefore, we establish a 1∶1 correlation between the shear stresses generated by the device (the agonist that activates platelets in this case) and the thrombin (the more relevant and universal coagulation marker) generation by the activated platelets. Platelet activation was normalized to maximum activation determined by sonication [9] and was reported for each VAD. One way ANOVA was used to statistically infer pair-wise differences in slope of the fitted lines (Platelet Activation Rate, PAR) between the original and optimized VAD design.

## Results

We report results from the numerical modeling of the two VADs followed by experimental measurements of platelet activity performed in the two VAD prototypes. The results have been reported in the following order: First, numerical modeling results of the two VADs are illustrated by the velocity vector flow field (freeze frames from FSI modeling) and platelet dispersion patterns before and after optimization. This is followed by detailed analysis of stress accumulation for thousands of platelet trajectories (designated as PDFs) that pass through the entire VADs (overall PDFs) and specific problematic regions of the VADs (region specific PDFs). We have illustrated specific examples of platelet trajectories and flow fields in both VADs that show optimization was indeed achieved. Following the numerical results, we report experimental platelet activation measurements performed in both VAD prototypes and the strong confirmatory correspondence to numerical findings indicating an order of magnitude lower thrombogenicity in the optimized VAD.

### Improved Platelet Dispersion in Optimized VAD

**Figure 5:**
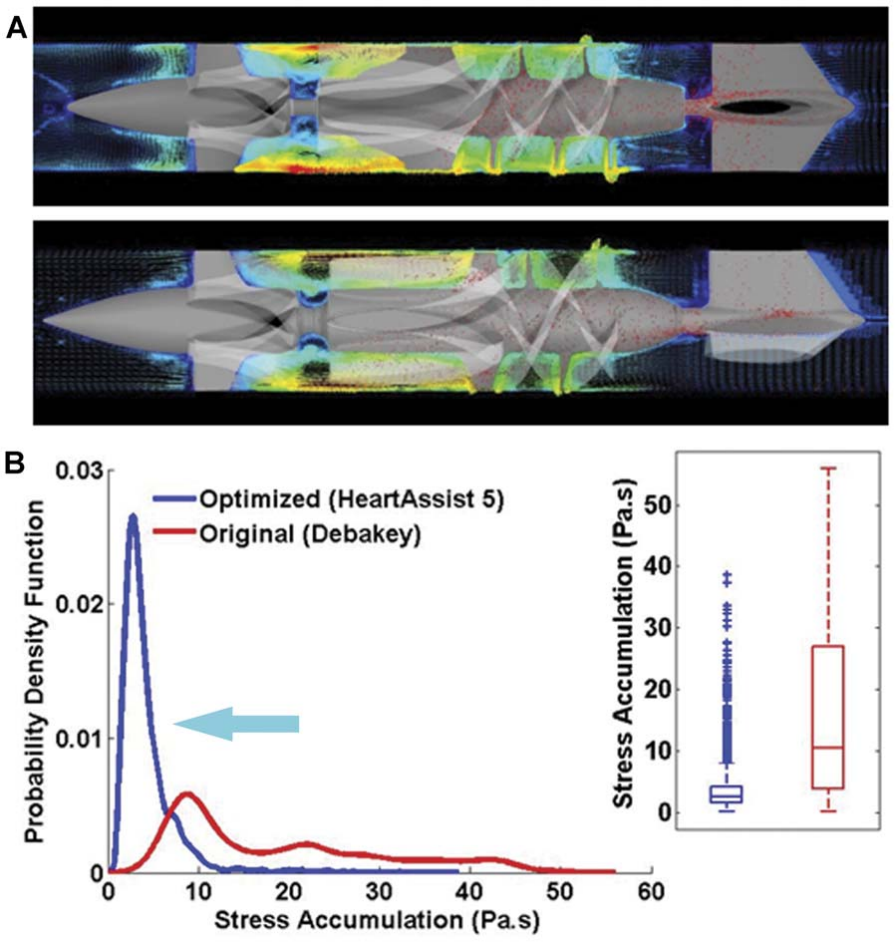
Numerical Analysis of the VAD prototypes. (**A**) Flow fields at the mid-cross section in both VAD designs (top: original VAD; bottom: optimized VAD). The platelet dispersion patterns (red) are superimposed on the velocity vector field in the mid cross-section. (**B**) Overall probability density functions (PDF) of stress accumulation (SA) for both VADs (red – original design; blue – optimized design). The *inset* shows the statistical distribution with its outliers.

A general depiction of the flow fields (platelet dispersion patterns superimposed on the velocity vector field) is shown in Figure 5A (top: original VAD; bottom: optimized VAD) at the mid-cross section in both VADs. This demonstrates how the new streamlined tapered rear hub with blood flow incoming through the 3 blade stator vanes eliminates the problem of the original design in which the inflow through the 4 blade stator was impinging against the hub shoulder and stagnating in that region. The platelet dispersion patterns showing transition from the inlet flow straightener to the inducer-impeller region are further illustrated in Figures S1 and S2 (original VAD (top); optimized VAD (bottom)). This demonstrates that the new impeller design with a steeper pitch (blade angle of attack) generates more favorable flow conditions that are less activating to the platelets, additionally indicating that the ‘time of flight’ of the platelets is shorter through the optimized design – reducing the duration platelets are exposed to elevated shear stresses (quantitative details appear in the PDF analysis below).

### Lower Overall Stress on Platelets in Optimized VAD

Stress accumulation (SA) was computed in both prototypes for 25 impeller rotations. The PDF was computed for an ensemble of 4,000–5,000 platelet trajectories passing through the two VAD designs, and was extrapolated and recalculated for 40,000 and 50,000 platelets respectively (to account for a more physiologically relevant platelet count) by applying bootstrapping statistics [12], [13]. The resulting PDF of the SA ranges distribution (the ‘thrombogenic footprint’ of the device) is shown in Figure 5B. A very significant shift (to the left) in the mean stress accumulation to lower values (*p*<0.01) was observed with the optimized design (mean SA ± SD: 3.5±3.3 Pa.s), as compared to the original design (mean SA ± SD: 16.2±14.4 Pa.s) – with the latter having a broader distribution (low kurtosis and larger standard deviation), thereby placing a very large number of platelets in the higher end of the SA spectrum as compared to the optimized design.

### Statistical analysis of PDFs and Outliers

Outliers in the statistical distributions that appear in Figure 5B (*inset*) are often indicative either of measurement error or that the population has a heavy-tailed distribution (like in our case, Log-normal distributions). In the former case one wishes to discard them or use statistics that are robust to outliers, while in the latter case they indicate that the distribution has high kurtosis (representing a very spiky distribution with a small standard deviation, similar to our distribution in Figure 5B – blue line) and that one should be very cautious in using tools that assume a normal distribution (in our analysis we have to use non-parametric statistics or transform the Log-normal distributions to Normal distributions). The statistical analysis indicates that the HeartAssist 5 distribution (blue line) in Figure 5B has high kurtosis (mean SA ± SD: 3.5±3.3 Pa.s) with a heavy tail of outlier SA values. On the other hand, the original DeBakey design (red line, Figure 5B) has a much broader distribution with low kurtosis and larger standard deviation (mean SA ± SD: 16.2±14.4 Pa.s). While the original DeBakey design clearly has less ‘outliers’, its distribution places a staggering amount of platelets in the higher end of the SA spectrum as compared to the optimized design. The statistical tools used indicate that the HeartAssist 5 distribution (blue line) has many outliers, but even when considering the outliers in this distribution, the range of the SA is much lower (highest SA value is approx. 40 Pa.s) than the original DeBakey design (highest SA value is approx. 60 Pa.s), and also has a much lower mean value. However, these outlier SA values could still lead to some undesirable platelet activation. Further analysis could include case specific analysis of these outliers that can be utilized for additional optimization of the VAD.

### Design Modifications in Optimized VAD Increases its Thromboresistance

**Figure 6:**
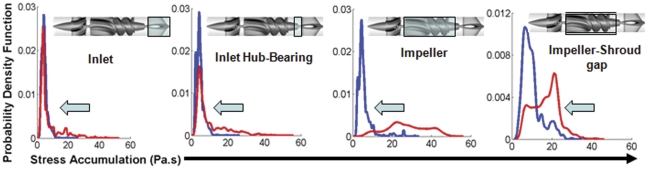
Design change *ROI* specific PDFs (left-right): inlet flow straightener, inlet hub and bearing, impeller, impeller tip-shroud gap and near shroud region. The *ROI* are indicated by boxed areas (*inset*).

**Figure 7:**
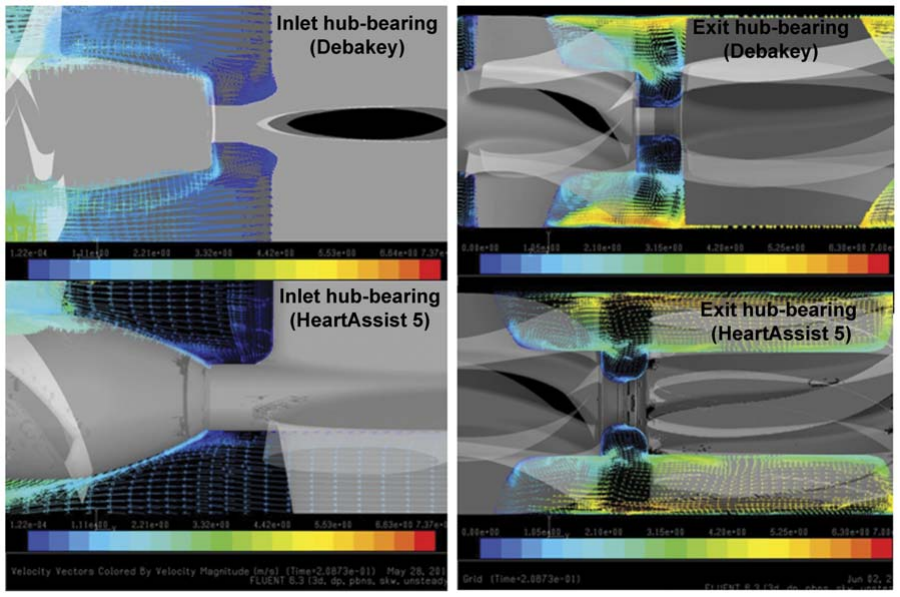
Velocity vectors (mid-plane) showing improved blood washing (reduced recirculation and stagnation) at the hub and bearing interfaces at both inlet (left, straightener-impeller interface) and exit (right, impeller-diffuser interface) for original (top) and optimized (bottom) VAD designs.

To delineate the specific regions implicated in higher overall thrombogenicity of the original and optimized VAD designs, we compared mean stress accumulation distributions specific to regions of design changes shown in Figure 2. Regions of Interest (*ROI*) were identified corresponding to these design changes and the PDFs of stress accumulation were calculated in these *ROI* (blue boxed areas in Figure 6 – *insets*; left-right): inlet flow straightener, inlet ball-in-cup bearing, impeller region, and impeller-shroud gap and near shroud region. A significant shift in mean SA to lower values was observed for the various *ROI* in all cases (Table 1). Specifically, as a consequence of design modifications to the impeller region, markedly fewer platelets were exposed to regions of high stress accumulation (10–50 Pa.s) in the optimized VAD. Similarly, the design optimization at the inlet flow straightener reduced exposure time of platelets to high stresses and eliminated flow stagnation that was observed in the original VAD design (Figure 7-left and Figure S1). A similar modification at the inducer-impeller interface depicted reduced recirculation and smaller vortices as compared to the original VAD design (Figure 7-right). Furthermore, in the optimized VAD design, fewer platelets (84%) passed through the *ROI* close to the shroud wall and gap between the impeller tip and the shroud (where the shear stresses are the highest), as compared to 94% for the original VAD design.

**Table 1:** Mean (± SD) of stress accumulation (SA) for platelet trajectories passing though regions of interest (*ROI*) in original and optimized VADs.

Design Change *ROI*	Original VAD SA (Pa.s)	Optimized VAD SA (Pa.s)
Impeller	27.1±13.1	5.1±4.4
Impeller-Shroud gap	15.4±8.2	8.8±5.8
Flow Straightener	7.1±8.2	3.9±2.2
Hub and Bearing	8.8±9.3	4.1±2.3

Results correspond to PDFs shown for each *ROI* in Figure 6.

### Stress Loading Waveforms Extracted from Hot-Spot Trajectories for DTE Experiments and their Correspondence to PDFs

**Figure 8:**
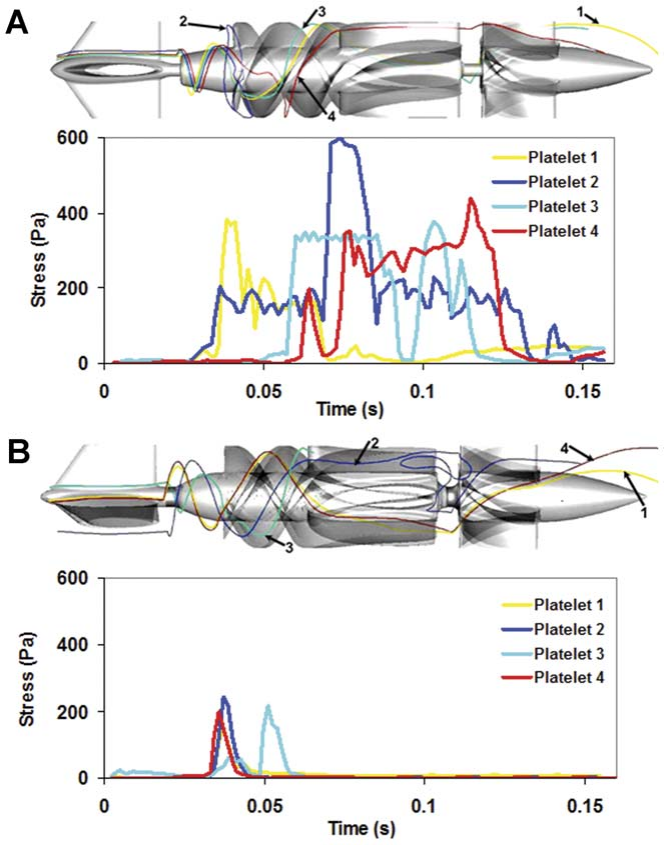
Markedly reduced stress accumulation of platelet trajectories in optimized VAD. Representative stress and exposure time for 4 platelet trajectories during their passage through the (**A**) original VAD (DeBakey) and (**B**) optimized VAD (HeartAssist 5) are shown.

The significantly lower SA observed in the optimized design PDF is further corroborated by analysis of typical hot-spot platelet trajectories (ones that expose platelets to elevated shear stresses for a duration that may activate them) during their passage through the VADs, and their resultant stress loading waveforms that are programmed into the HSD to emulate design dependent extreme flow conditions in the devices that may activate platelets. It clearly illustrates that platelets flowing through the original VAD experience elevated stresses and longer exposure times, in contrast to platelets flowing through the optimized design (Figure 8). Representative platelet stress waveforms extracted from hot-spot trajectories show that platelets flowing through the original VAD (Figure 8A) experience elevated stresses (200–600 Pa) and longer exposure times, in contrast to platelets flowing through the optimized design (Figure 8B; peak stress below 200 Pa and significantly lower exposure times to high stresses). For example, platelet 2 (Figure 8A) is trapped at the impeller-inducer interface and experiences the highest stress (∼600 Pa). From these 4 trajectories that correspond to platelets flowing through the highest shear stress regions (hot-spots), it is easy to infer that the stress accumulation is substantially minimized for the optimized design. This is further reiterated by the PDFs themselves that represent the overall activation potential of the platelet bulk while passing through the VAD (i.e., the statistical distribution of final stress accumulation values reached in each of the multiple trajectories).

### Order of Magnitude Lower Platelet Activation Rates Measured in the Optimized VAD Prototype

**Figure 9:**
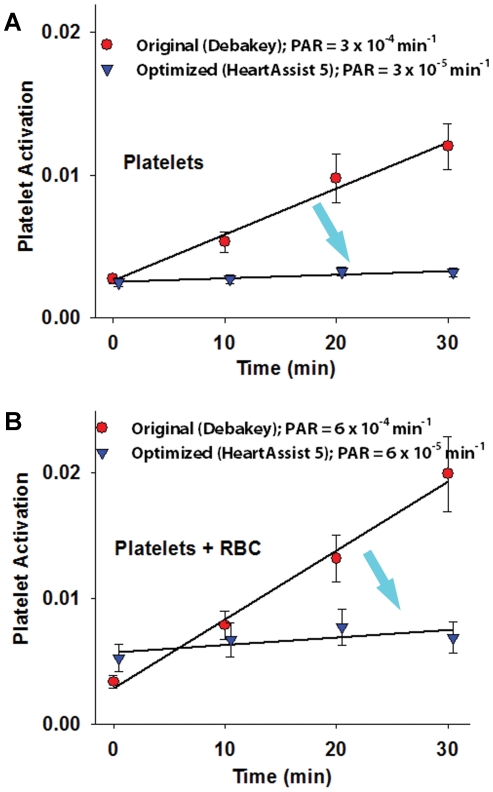
Platelet activation measurements in VAD prototypes. (**A**) Platelet Activation Rate (PAR) in the original and optimized VAD prototypes measured in a flow-loop with the modified prothrombinase assay (platelet experiments only, n = 10, mean ± SEM plotted for each point) (**B**) Platelet Activation Rate (PAR) in the original and optimized VAD prototypes measured in a flow-loop with the modified prothrombinase assay (platelets and red cells experiments, n = 5, mean ± SEM plotted for each point). An order of magnitude lower platelet activation rate-slope (PAR) is achieved by the optimized design in both cases.

Platelet activation was measured with the modified prothrombinase assay [10] in identical flow loops (Figure 4) for 30 min for both VAD prototypes operating at: cardiac output (flow rate) of 4 L/min; pressure rise across the VADs of 70–80 mm Hg; and impeller rotation speed of 9500 rpm, as described in Materials and Methods. Experiments were conducted with platelets alone (n = 10 donors) and then repeated with purified platelets and added autologous red blood cells (RBC; n = 5 donors). In both cases, respectively, an order of magnitude reduction in Platelet Activity Rates (PAR – the slope of the platelet activation linear fit) was observed (3×10^−4^ min^−1^ and 6×10^−4^ min^−1^ for the original VAD and 3×10^−5^ min^−1^ and 6×10^−5^ min^−1^ for the optimized VAD – Figure 9A (platelets only) and Figure 9B (platelets and red blood cells), *p*<0.01). This result was in full agreement with the numerical results of the significant shift towards lower SA values indicated by the PDFs of the optimized design as compared to the original design, as illustrated previously.

## Discussion

In the NHLBI 2004 Working Group report for Next Generation VADs for Destination Therapy, a major recommendation was to develop improved antithrombotic therapies and device technologies to reduce the vexing problem of thromboembolic events, based on successful computational and experimental fluid dynamic studies within prosthetic heart valves [18]. Following this critical recommendation, we developed and validated the DTE methodology in pilot studies to demonstrate the effect of sensitive design parameters on the thrombogenic potential of components of MCS devices, as well as stand-alone prosthetics (two bileaflet MHVs – the St. Jude Medical (SJM) Regent MHV, and the ATS MHV). With DTE, it was demonstrated that the ATS open pivot hinges offer a better design solution than the SJM recessed hinges [13], and that the ATS design can be significantly optimized further [12]. In this study we have extended the application of the DTE methodology to a complex MCS device – the rotary VAD, and demonstrated the universal nature of this optimizing methodology for significantly improving the thrombogenic performance of blood recirculating cardiovascular devices.

Advantages of continuous flow blood pumps over pulsatile first generation VADs, include miniaturized size, increased mechanical durability and hemodynamic efficiency, reduced cost, and improved bridge to transplant rates [19]. Previous studies on thromboresistance optimization of such continuous flow VADs have investigated the parametric effects of features such as: impeller tip-shroud gap clearances [20], [21], diffuser inlet angles [22], geometric profiles of flow straightener, impeller and diffuser [22], [23], [24], impeller-inducer (moving components of the VAD) angular alignments and axial clearances and impeller rotation rates [25] with CFD, particle image velocimetry and RBC hemolytic testing methods. Significant limitations of these studies as thromboresistance optimization tools are due to analyses performed only in the numerical domain and/or conclusions based on hemolysis damage models (hemolysis occurs at shear levels one order of magnitude greater than those required to activate platelets [26]). DTE, on the other hand is a comprehensive and universal optimization strategy that offers direct correlation with physical thrombosis and coagulation markers. Its clear advantage over previous approaches is demonstrated in the current application for VAD design optimization, where the effects of specific design changes on platelet activation potential are quantitatively analyzed and directly correlated to measured thrombogenic markers.

The motivation for the design changes in the DeBakey VAD stem from several preclinical and clinical studies conducted with the initial VAD design [7], [8], [27], [28], [29], [30] that identified thrombogenic regions (inlet hub and bearing and flow straighteners) within the VAD. For instance, thrombus formation was observed in *ex vivo* studies at the inlet hub and bearing interface and the inlet [7]. Corresponding design changes were made (Figure 2) to the above components to reduce backflow, vortex formation and flow stagnation, surface contact area with the blood, and to facilitate a smoother transition from the straightener vanes to the inducer-impeller region.

Stress-loading histories (waveforms), of platelets flowing through distinct regions within the two VAD designs, were computed and correlated with platelet activation measurements performed in the two prototypes that quantify the overall device thrombogenicity. The PDFs of the stress accumulation (‘thrombogenic footprint’ of the overall device (Figure 5B)), as well as the PDFs of specific regions of interest such as the inlet, the inlet hub-bearing interface, the impeller, and the impeller-shroud gap near the shroud (Figure 6), clearly indicate that the intended design changes significantly reduced VAD thrombogenicity (design optimization significantly shifted the SA distributions towards lower values, i.e., to a significantly reduced thrombogenic potential). The improved hemodynamics of the optimized design are evident using more traditional comparison of the flow fields of the two designs, which depict the elimination of stagnation regions and improved washing properties in the inlet and exit hub-bearing (Figure 7) of the optimized VAD.

A closer examination of these improved hemodynamics patterns is facilitated by the two-phase flow simulation that reveals and quantifies the platelets dispersion patterns when flowing through the devices (Figure S2). The number of platelets traversing the near shroud region and impeller tip-shroud gaps is reduced by 10% in the optimized VAD design, translating to a significantly lower number of platelets exposed to the higher stress loading along such hot-spot trajectories. This clearly indicates that the optimized design entrains platelets towards the rotating core, away from the elevated stresses regions of the gap clearance. The stress accumulation PDF for the *ROI* in the gap between the rotor blade tip and the shroud (housing) shows a 2-fold reduction in stress accumulation. This also follows the overall 5-fold lower stress accumulation in the impeller region (Figure 6) wherein a series of design changes significantly reduces the platelet residence time and surface contact area, thereby minimizing the peak stresses and exposure times. This is further corroborated by the platelet stress waveforms extracted from hot-spots trajectories comparing the original VAD (Figure 8A) with the optimized VAD (Figure 8B), which very clearly demonstrate how the optimization process resulted in far lower stress-exposure conditions for platelets flowing in the optimized VAD.

Measurement of thrombin represents the actual contribution of platelets to the coagulation cascade final product of blood clot formation. Traditional platelet activation surface markers offer a less universal marker as they point only to specific aspects of the platelet activity – not necessarily to the clotting product (thrombin cleaves soluble fibrinogen into insoluble fibrin – the building block of blood clot formation). Our focus in this manuscript is to illustrate a methodology applied to design optimization of a VAD with the goal of minimizing the occurrence of pathological fluid shear stresses that lead to platelet activation (eventually leading to coagulation). In our experimental studies, we therefore use purified blood platelets over whole blood or plasma experiments since with the former choice we are able to isolate and measure the direct effect of pathological shear induced damage on platelets and their capacity to generate thrombin. This 1∶1 correspondence measured with the most relevant coagulation marker (thrombin) offers a direct comparison between flow-induced thrombogenicity of different device designs. This is therefore our method of choice for measuring the thrombogenic potential of blood recirculating devices, and our group established its validity over the years in addition to successfully demonstrating a strong correspondence between thrombin measured with the PAS method and between platelet surface activation markers (P-selectin) [31].

While it is clear in the clinical domain that patients, for overall clinical safety will remain on anticoagulants, i.e. Vitamin K antagonists and antiplatelet medication, i.e. Aspirin (ASA), the extent, complexity and dose of these regimens may very likely be reduced using DTE. Reduction of the extent of antithrombotic medication that a patient is required to take will also help reduce the significant side effect burden associated with many of these medications, e.g. gastrointestinal bleeding and hemorrhagic stroke. This however is not the focus of our current study.

In summary, the efficacy and broad applicability of the DTE methodology for achieving thromboresistance optimization of MCS devices was demonstrated in this study in a complex MCS device — a rotary VAD. The DTE proof-of-concept applicability was established by comparing the thrombogenicity of a continuous flow rotary VAD before and after optimization. Specifically, an order of magnitude lower Platelet Activity Rate (PAR) was measured in the optimized VAD prototype as compared to measurements in the original VAD — correlating to the very significant overall shift in the mean stress accumulation (SA) to lower values (the device ‘thrombogenic footprint’ predicted by the DTE).

The DTE optimization methodology described herein demonstrates the use of advanced numerical simulations combined with experimental techniques for quantifying measures that are directly relevant to thrombosis and coagulation markers in devices. It offers an unparalleled degree of freedom to test and redesign any type of MCS device as it is device independent, i.e., it emulates the device hemodynamics and enables measuring the resultant device thrombogenicity without the need to build the redesigned prototype first – rather following its optimization. It is envisioned that the DTE approach will become essential in guiding the design and enhancing the safety and effectiveness of many therapeutic implant devices placed in the blood stream. In addition to reducing R&D costs, DTE may prevent unfortunate situations in which devices need to be recalled or clinical trials stopped, due to unacceptable levels of clinical thromboembolic events – situations which would be catastrophic to patients, with devastating financial costs to device manufacturers and society as well. These savings will ultimately be passed on to patients and will further aid in reducing the escalating costs of healthcare.

## Supporting Information

Figure S1 **FSI simulation results showing platelet (red) passage and velocity vector flow fields (mid cross-section) in original (top) and optimized (bottom) VAD prototypes.**
(GIF)

Figure S2 **Isometric view representation of platelet (red) dispersion patterns in the original (top) and optimized (bottom) VAD prototypes.**
(GIF)
