# Supplementary figures and images for: Device Thrombogenicity Emulation: A Novel Method for Optimizing Mechanical Circulatory Support Device Thromboresistance
Source: PLoS One. 2012 Mar 2;7(3):e32463. doi: 10.1371/journal.pone.0032463 (PMC3292570; doi:10.1371/journal.pone.0032463)

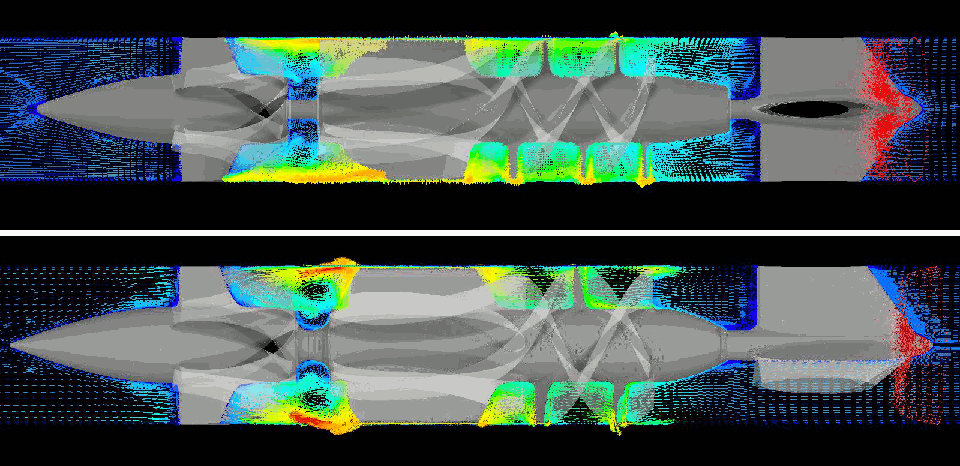

Supplement: Figure S1 — FSI simulation results showing platelet (red) passage and velocity vector flow fields (mid cross-section) in original (top) and optimized (bottom) VAD prototypes. (GIF) [file pone.0032463.s001.gif]

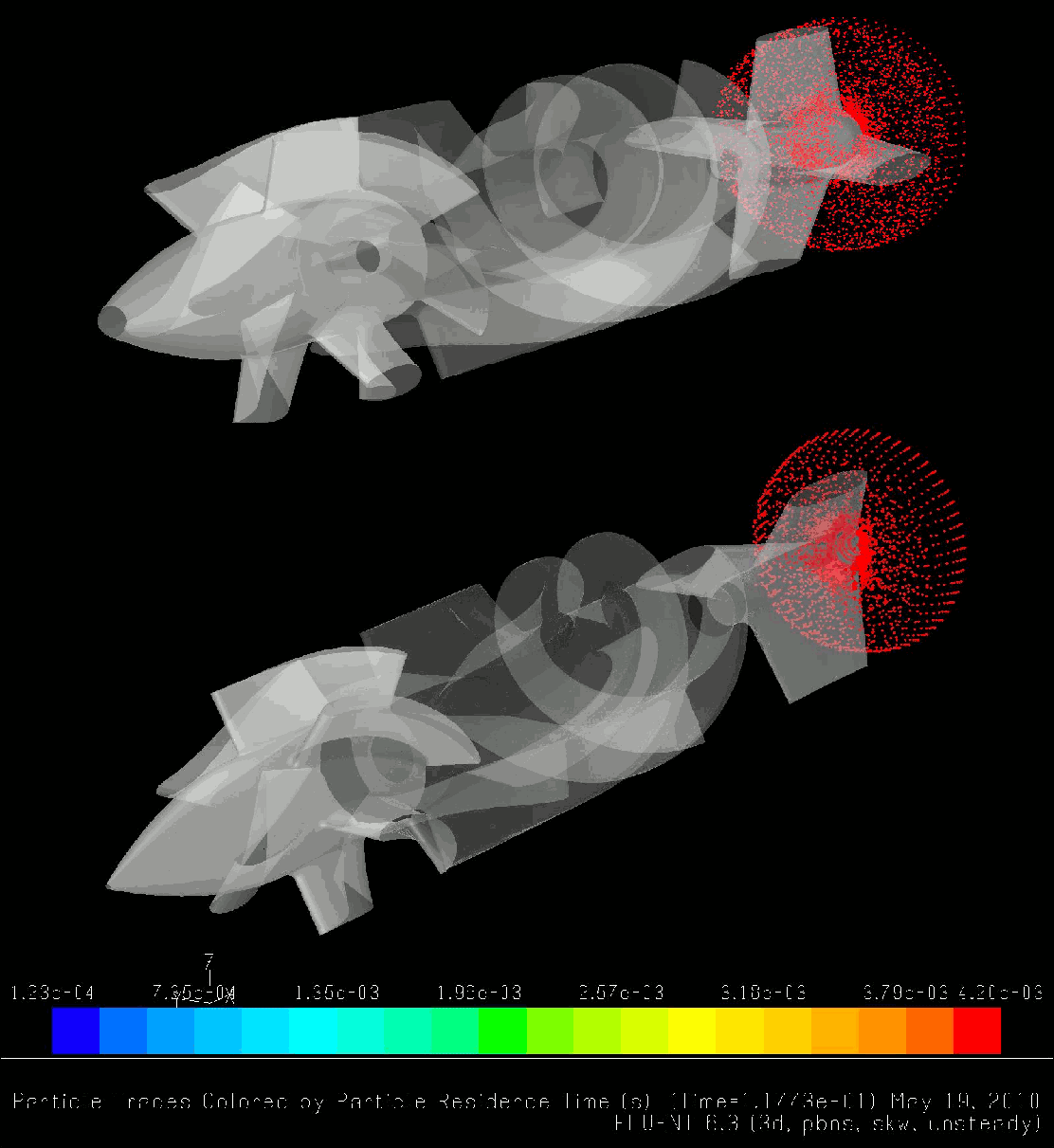

Supplement: Figure S2 — Isometric view representation of platelet (red) dispersion patterns in the original (top) and optimized (bottom) VAD prototypes. (GIF) [file pone.0032463.s002.gif]
